# Stacking-Order-Dependent Electronic Properties of MoTe_2_/WSe_2_ Moiré Bilayers

**DOI:** 10.1021/acs.nanolett.6c02425

**Published:** 2026-10-26

**Authors:** Zhongdong Han, Wenjin Zhao, Eegene Clara Chung, Chia-Hao Lee, Zui Tao, Zhengchao Xia, Yichi Zhang, Yiyu Xia, Jekwan Lee, Bowen Shen, Ariana Ray, Yu-Tsun Shao, Tingxin Li, Shengwei Jiang, Yihang Zeng, Kenji Watanabe, Takashi Taniguchi, David A. Muller, Kin Fai Mak, Jie Shan

**Affiliations:** † Laboratory of Atomic and Solid State Physics, 5922 Cornell University, Ithaca, New York 14853, United States; ‡ Department of Physics, 5922 Cornell University, Ithaca, New York 14853, United States; § 375070 Max Planck Institute for the Structure and Dynamics of Matter, Hamburg 22761, Germany; ∥ Kavli Institute at Cornell for Nanoscale Science, Ithaca, New York 14853, United States; ⊥ School of Applied and Engineering Physics, 5922 Cornell University, Ithaca, New York 14853, United States; # 52747 National Institute for Materials Science, Tsukuba 305-0047, Japan

**Keywords:** TMD moiré bilayers, stacking order, electronic properties, SHG, STEM, interlayer hybridization

## Abstract

Transition metal dichalcogenide (TMD) moiré bilayers have realized a wide range of strongly correlated and topological phenomena. The physics in these materials is often sensitive to the interlayer stacking order. Polarization-resolved optical second harmonic generation (SHG) is the most used technique for stacking order characterization but unverified for most heterobilayers. Here we calibrate the optical SHG for angle-aligned MoTe_2_/WSe_2_ bilayers by the scanning transmission electron microscopy (STEM). We directly compare the transport and magnetic properties and the electronic phase diagram for two distinct stacking orders. With the calibrated stacking order assignment, we clarify the interpretation of earlier results, including the nature of the Chern insulator, mechanism of an electric-field-tuned metal–insulator transition at half band filling, and the Kondo lattice physics. Our work provides a consistent picture of the relation between the stacking order and the electronic properties of MoTe_2_/WSe_2_ moiré bilayers.

## Graphical abstract

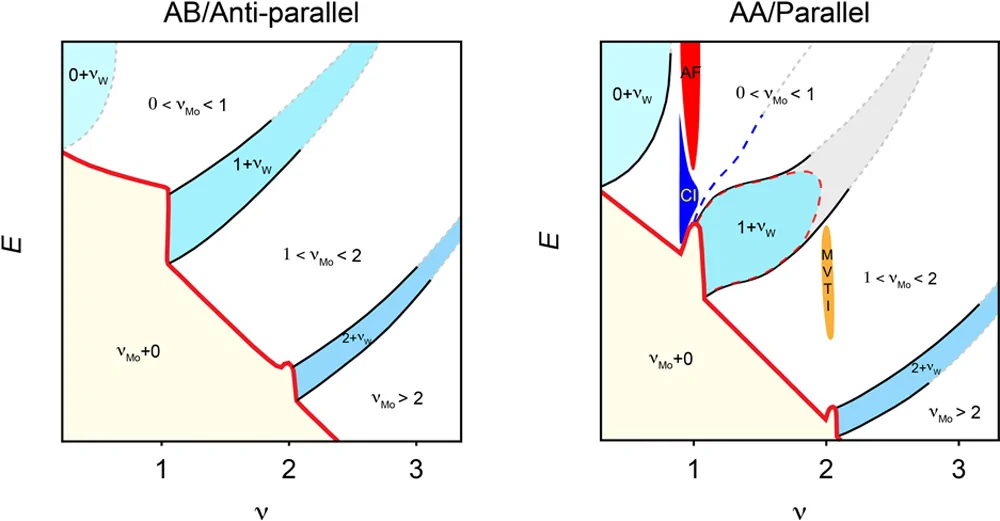

Semiconductor moiré materials have emerged as a highly tunable platform for studying electronic correlations and topology. A wide range of phenomena, including Mott and Wigner-Mott insulators,^1–14^ kinetic antiferromagnetism and spin polarons,^15–19^ Kondo lattice physics,^20–28^ and the quantum anomalous Hall^29–36^ and quantum spin Hall effects,^29,34,37,38^ has been demonstrated in one system--angle-aligned MoTe_2_/WSe_2_ vertical heterobilayers.^39–42^ Many of these phenomena are sensitive to stacking order, that is, whether the two transition metal dichalcogenide (TMD) monolayers are parallel (AA-stacking) or antiparallel (AB-stacking) to each other (Figure 1a,b).

**Figure 1:**
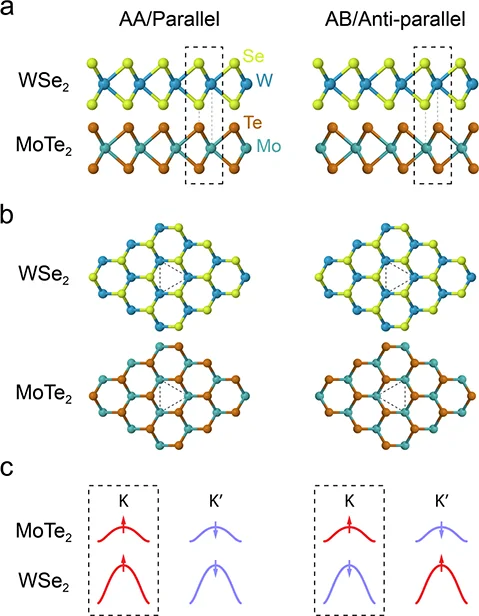
Stacking order and moiré bands of angle-aligned MoTe_**2**_/WSe_2_ bilayers. a, Side view. b, Top view. The atoms are shown in blue (W), green (Se), cyan (Mo) and brown (Te). c, Spin-resolved topmost moiré valence bands from MoTe_2_ and WSe_2_. Left: AA-stacking with two layers parallel to each other; right: AB-stacking with two layers antiparallel to each other. In each valley (K/K′), electron spins (arrows) in the two layers are parallel (AA) or antiparallel (AB) due to spin-valley locking in monolayer TMDs. Interlayer hybridization is spin allowed for AA-stacking and spin forbidden for AB-stacking.

Angle-aligned MoTe_2_/WSe_2_ bilayers form a moiré lattice with period *a*_M_ ≈ 5 nm because of the 7% lattice mismatch between the two TMDs. The electronic states of interest are originated from the valence band in the K/K′ valley of monolayer MoTe_2_ or WSe_2_, where the presence of a strong Ising spin–orbit interaction creates a large spin splitting in each valley and the spin and valley degrees of freedom are locked.^41,43^ Because the moiré momentum is negligible compared to the atomic scale momentum, moiré bands can hybridize only with those from the same valley (Figure 1c). The spin-valley locking renders the moiré band structure stacking dependent: interlayer hybridization is spin allowed for AA-stacking and spin forbidden for AB-stacking.^31^ This stacking-dependent interlayer hybridization, however, poses significant challenges in understanding several earlier experimental results, including the nature of the Chern insulator, mechanism of an electric-field-tuned metal–insulator transition at half band filling, and the Kondo lattice physics.^1,20,29,37^

In all previous experiments, stacking order in angle-aligned MoTe_2_/WSe_2_ bilayers was determined by the polarization-resolved optical second harmonic generation (SHG).^44,45^ Specifically, monolayer TMDs lacking inversion symmetry allow for a second-order response; the second harmonic (SH) dipole moment is uniquely determined by the crystal’s symmetry.^46–48^ In angle-aligned bilayers, the SH dipole from each monolayer is expected to be in-phase (out-of-phase) for AA-(AB-)­stacking, leading to constructive (destructive) interference of the SH radiation. This convenient optical technique has been extensively studied in natural TMD bilayers and multilayers^46–48^ but remained unverified for heterobilayers except WS_2_/WSe_2_ (refs 44 and 45 ). In this work, we directly determine the stacking order in angle-aligned MoTe_2_/WSe_2_ bilayers by the scanning transmission electron microscopy (STEM) and establish a correlation between the stacking order and SHG. Contrary to homobilayers, we observe destructive interference in SHG for AA-stacked MoTe_2_/WSe_2_ bilayers with excitation at 800 nm. Based on the revised stacking order assignment, we present a consistent picture of stacking-dependent electronic phase diagrams of the material.

## Structure Characterization

We directly determine the structure of MoTe_2_/WSe_2_ bilayers by atomic-resolution imaging using STEM. The annular dark-field (ADF) image intensity scales approximately with *Z* ^1.7^, where *Z* is the atomic number (ref.^49^), and the contributions from overlapping chalcogen atoms are summed (Table 1). The atoms in each TMD monolayer can be clearly identified: the W sites appear about two times brighter than the Se_2_ sites in WSe_2_, and the Te_2_ sites are about three times brighter than the Mo sites in MoTe_2_. But the W and Te_2_ sites have similar intensities, making accurate identification of the atoms and consequently stacking order in heterobilayers difficult.

**Table 1:** ADF-STEM Intensity Scaling with Atomic Numbers in MoTe_2_/WSe_2_ Bilayers^a^

element	atomic number *Z*	HAADF contrast ∼ *Z* ^1.7^	total contrast (a.u.)
Mo	42	575	575
W	74	1505	1505
Se	34	401	802 (Se_2_)
Te	52	826	1652 (Te_2_)

a The high-angle annular dark-field (HAADF) contrast is approximately proportional to *Z* ^1.7^ (ref 49). The contributions from overlapping chalcogen atoms are summed (a.u.: arbitrary unit).

We designed a special sample geometry to unambiguously determine the stacking order in MoTe_2_/WSe_2_ bilayers by STEM and correlate it with SHG (see Methods for details on the sample fabrication and STEM and SHG measurements). Figure 2a,b shows two MoTe_2_/WSe_2_ samples angle aligned based on SHG. The MoTe_2_ and WSe_2_ monolayers in these samples were cut from the same flakes and one MoTe_2_ layer was rotated by 180° relative to the other. The two bilayer samples are thus guaranteed to have different stacking orders. Figure 2d,e shows the ADF-STEM images of the isolated WSe_2_ and MoTe_2_ monolayer regions of sample 1 (Figure 2a). Intensity profile analysis of these images (Figure S1) clearly identifies the metal and chalcogen atoms. We determine the overlapped region to be AB-stacked based on the relative orientations of the metal sublattices (denoted by yellow dashed lines) in the isolated monolayer regions. Similarly, we determine sample 2 (Figure 2b) to be AA-stacked based on the ADF-STEM images in Figure 2g,h (see Figure S2,3 for STEM images of the bilayer regions for further confirmation of the stacking order assignment).

**Figure 2:**
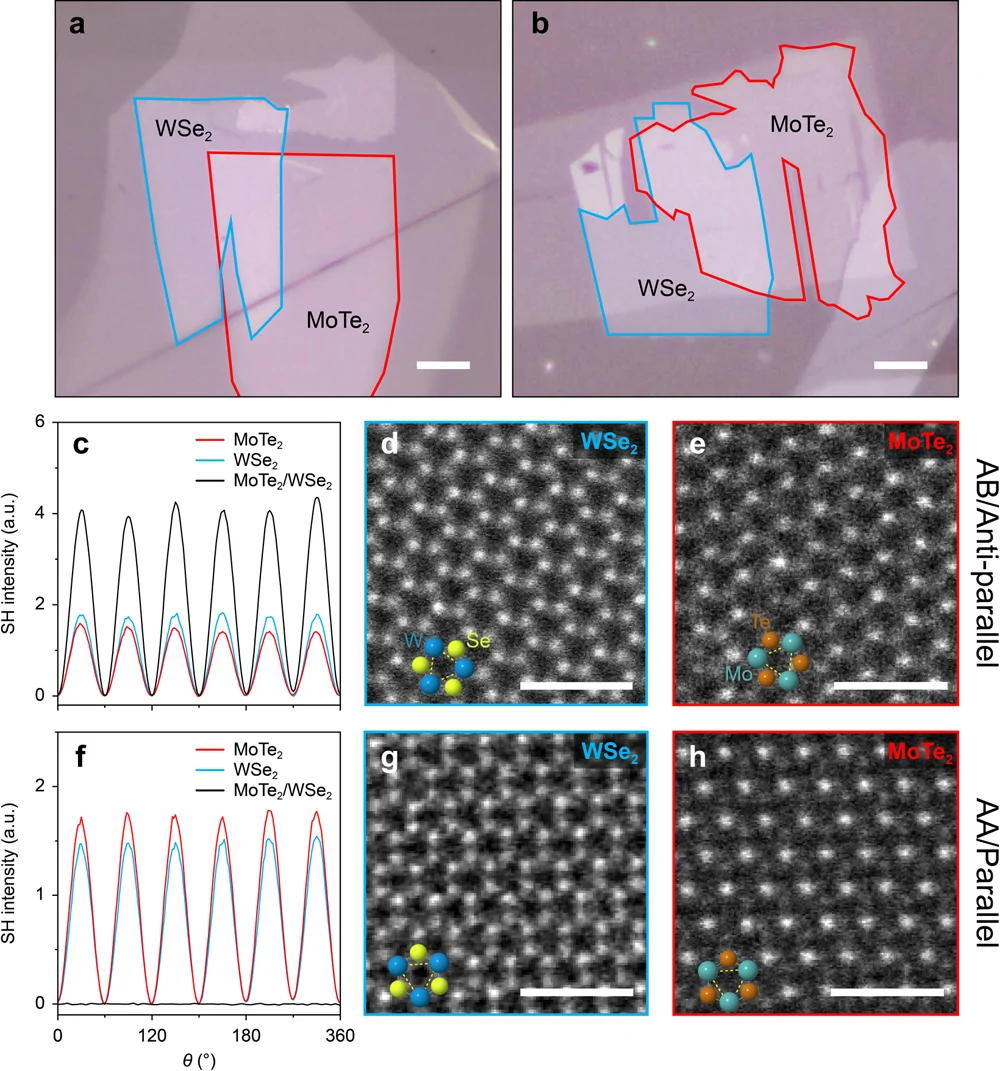
Stacking order determination. a, b, Optical images of angle-aligned MoTe_2_/WSe_2_ sample 1 (a) and sample 2 (b) on PC stamps. The WSe_2_ and MoTe_2_ layers are outlined in blue and red, respectively. Scale bars are 5 μm. The WSe_2_ (MoTe_2_) layers were cut from the same flake. A 180° rotation was introduced in the two MoTe_2_ layers. SHG with excitation at 800 nm and STEM measurements were performed on both the isolated monolayer regions and the bilayer region (Methods). c–e, Characterization of sample 1. The SH intensity (c) exhibits a 6-fold rotational symmetry as a function of the excitation polarization angle. The bilayer response is 2–3 times of that of each monolayer. ADF-STEM images from the WSe_2_ (d) and MoTe_2_ (e) monolayer regions identify the atoms (colored balls) and the metal sublattices (marked by yellow dashed lines). Scale bars are 1 nm. f–h, Characterization for sample 2. The SH intensity for the bilayer nearly vanishes.

We correlate the SHG with stacking order by performing SH measurements on both the isolated monolayer regions and the bilayer region (Figure 2c,f). We used the reflection geometry with normal incidence for excitation at 800 nm. The SH intensity in cross-polarization exhibits a 6-fold symmetric response as a function of the linear polarization angle of the excitation beam, or equivalently, the crystal’s azimuthal angle. Such a response is consistent with the 3-fold rotational symmetry of monolayer TMDs^46–48^ and agrees with previous reports.^1,29^ The SH intensity is comparable for monolayer MoTe_2_ and WSe_2_. But surprisingly, it nearly vanishes for the AA-stacked sample (Figure 2f) and is about 2–3 times of that of each monolayer for the AB-stacked sample. The imperfect constructive interference in the latter is likely caused by the finite optical absorption at the SH wavelength (400 nm) and by stacking-induced modifications on the optical response.

The result highlights that the SH response is a frequency-dependent complex function and can be significantly altered when either the fundamental or SH radiation is close to an electronic transition. Given the large difference in two TMDs’ band gaps and the 800 nm excitation lying between the two gap values, a phase shift between the SH dipole moments from two layers in AA-stacked bilayers is generally expected. This is further supported by the excitation wavelength dependence of SHG from another AA-stacked MoTe_2_/WSe_2_ bilayer (Figure S4). We observe destructive interference of the SH radiation for all three excitation wavelengths (800 nm, 1100 and 1220 nm), with most complete cancelation at 800 nm. The results are fully consistent with a recent detailed wavelength-dependence study on the same material.^50^ The *ab initio* calculations in ref.^50^ further suggest a π-phase shift in the SH response of the two TMD layers due to their distinct two-photon resonances.

## Electronic Phase Diagrams

Equipped with the calibrated SHG, we study the correlation between the stacking order and the electronic phase diagram in dual-gated MoTe_2_/WSe_2_ devices (whose stacking orders were characterized by SHG only) by combining the electrical transport and magnetic circular dichroism (MCD) measurements. The latter characterizes the magnetic properties of the bilayers. (See Methods and Figure S5 for stacking-order-dependent exciton hybridization). The two gates allow independent control of the lattice filling factor (ν) of holes in the bilayer and the electric field (*E*) perpendicular to the sample plane. Compared to earlier works, we used thinner hexagonal boron nitride (hBN) gate dielectrics to access higher breakdown fields and wider phase diagrams.^22^ Unless otherwise specified, the sample temperature was 1.6 K. See Methods for details on device fabrication and electrical and magneto-optical measurements.

The results for an AB-stacked MoTe_2_/WSe_2_ bilayer are shown in Figure 3. Figure 3a,b shows the longitudinal (*R*_*xx*_) and Hall (*R*_*xy*_) resistances, respectively, as a function of ν and *E* under a perpendicular magnetic field *B* = 14 T. The resistance maps are dominated by stripes, originating from the Shubnikov-de Haas (SdH) oscillations in the WSe_2_ layer, which has a more dispersive moiré valence band and higher hole mobility. (Substantially weaker SdH oscillations in the MoTe_2_ layer can also be discerned especially in the region of 1 < ν < 2). Diagonal stripes show that both layers are charge compressible and the partition in doping is tunable by electric field. In contrast, vertical stripes show that the MoTe_2_ layer is incompressible and the hole density in the WSe_2_ layer is independent of electric field.

**Figure 3:**
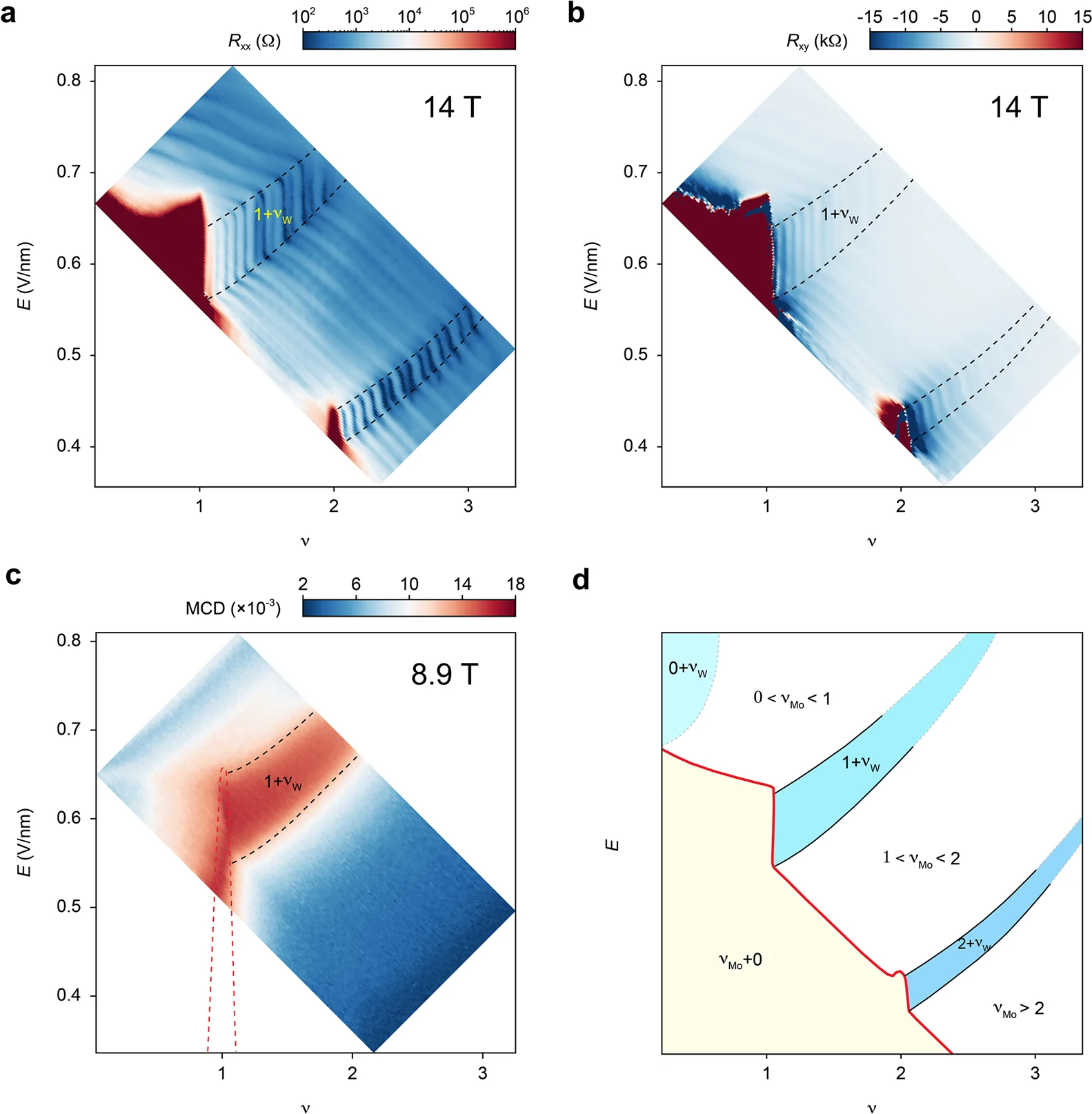
Electronic phase diagram for an AB-stacked device. Data are obtained from a device whose stacking structure is characterized by SHG only. a, b, Longitudinal resistance *R*_*xx*_ (a) and Hall resistance *R*_*xy*_ (b) as a function of ν and *E* at *T* = 1.6 K and *B* = 14 T. Regions (1 + ν_W_) and (2 + ν_W_) (enclosed by dashed lines) exhibit vertical stripes. c, MCD as a function of ν and *E* at *T* = 1.6 K and *B* = 8.9 T. The Mott insulating state at ν_Mo_ = 1 and region (1 + ν_W_) show a large MCD response and are marked by red and black dashed lines, respectively. d, Schematic phase diagram. Regions are labeled by filling factors in the WSe_2_ and MoTe_2_ layers.

We construct the electrostatic phase diagram in Figure 3d from the transport data. In the yellow region (ν_Mo_ + 0, left to the red solid line), only the MoTe_2_ layer is doped and the WSe_2_ layer is charge neutral (MoTe_2_/WSe_2_ is a type I heterojunction at *E* = 0 with the MoTe_2_ band gap lying entirely inside the WSe_2_ band gap). Here ν_Mo_ and ν_W_ denote the hole filling factors of the MoTe_2_ and WSe_2_ layers, respectively. The resistance in this region is generally high, likely due to the flat MoTe_2_ Hubbard bands, which favor charge localization. The other limit is the high *E*-field region (0 + ν_W_), where the WSe_2_ layer is doped and the MoTe_2_ layer is charge neutral. We could not access this region experimentally due to dielectric breakdown. In the two middle blue regions, the stripes are vertical, showing that the Fermi level is inside a gap of the MoTe_2_ layer—a Mott gap for (1 + ν_W_) and a moiré band gap for (2 + ν_W_)—and only the WSe_2_ layer hosts itinerant holes. The rest phase regions bear generic filling factors.

The constructed phase diagram is supported by the magnetic response of the bilayer. Figure 3c is the spectrally averaged MCD over the attractive polaron resonance of MoTe_2_ as a function of ν and *E* at *B* = 8.9 T. Strong MCD response is observed mainly around ν_Mo_ = 1 (denoted by red dashed lines) and in region (1 + ν_W_, marked by black dashed lines). This is consistent with the MoTe_2_ layer being a Mott insulator at half band filling; the spins after charge localization (i.e., local magnetic moments) are highly polarizable, giving a strong MCD response^44^ relative to the other phase regions.

The corresponding results for an AA-stacked MoTe_2_/WSe_2_ bilayer are shown in Figure 4 (see Figure S6,7 for comparison of resistance and MCD at *B* = 0). They are consistent with previous reports.^20,29,37^ The resistance maps (Figure 4a,b) share some similarities with those of the AB-stacked sample but are substantially richer. Similarly, we observe vertical resistance stripes in regions (0 + ν_W_), (1 + ν_W_) and (2 + ν_W_), and field-dispersive stripes in region (1 < ν_Mo_ < 2). On the other hand, we find vertical instead of diagonal stripes in region (0 < ν_Mo_ < 1) and new insulating states at ν = 1 and ν = 2. Particularly, the insulating state at ν = 1 for the intermediate *E*-fields, exhibiting vanishing *R*_*xx*_ and quantized *R*_*xy*_, is a Chern insulator (Figure 4d). In the presence of finite interlayer hybridization, the layer-resolved filling factors (ν_Mo_ and ν_W_) become approximate.

**Figure 4:**
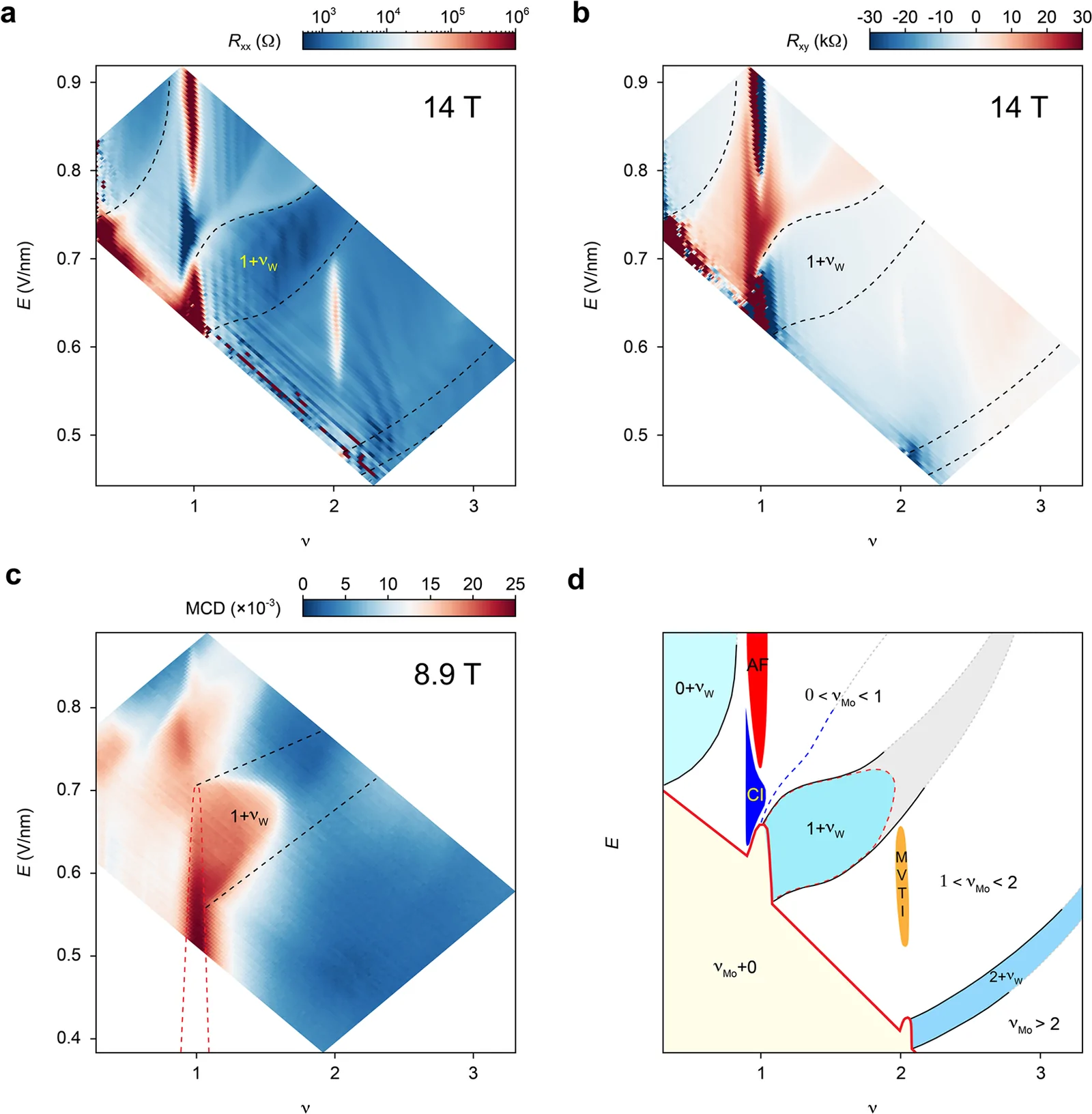
Electronic phase diagram for an AA-stacked device. Data are obtained from a device whose stacking structure is characterized by SHG only. Same as Figure 3 for an AA-stacked MoTe_2_/WSe_2_ bilayer. New states emerge in the phase diagram (d), including a Chern insulator (CI)^29^ and an antiferromagnetic (AF) insulator^68^ at ν = 1 and a mixed-valence topological insulator (MVTI)^22^ at ν = 2. The blue dashed line in region (0 < ν_Mo_ < 1) separates two Fermi surfaces: one with *R*_*xy*_ > 0 and another with *R*_*xy*_ < 0 and vertical SdH stripes; the latter has a small Fermi surface with doping counted from ν = 1. The red dashed line within region (1 + ν_W_) separates a light (blue-shaded) and heavy (gray-shaded) Fermi liquid. The former shows strong MCD.

The MCD map under *B* = 8.9 T (Figure 4c) also shows some similarities and differences between the two stacking orders. Specifically, a strong MCD response is also observed around ν_Mo_ = 1 and in region (1 + ν_W_) for the AA-stacked sample, where the MoTe_2_ layer is a Mott insulator. However, the strong MCD response in region (1 + ν_W_) terminates at an arc-shaped boundary, which expands into higher fillings with increasing magnetic field,^51^ whereas for the AB-stacked sample, strong MCD persists throughout the entire region. In addition, strong MCD is observed around ν = 1 for the intermediate *E*-fields, in accord with the Chern insulator.

The results in Figure 3,4 are consistent with the stacking dependent interlayer hybridization. The most important difference between the two stacking orders is how the SdH oscillations behave in regions with generic fillings. The AB-stacked samples have negligible interlayer hybridization. Therefore, the Landau levels in two layers are decoupled; both can be populated by gating and shifted in energy by electric field; the SdH stripes are diagonal in regions with generic fillings. Conversely, the AA-stacked samples have finite interlayer hybridization, weakening the electric-field effects. The Landau levels are layer hybridized in region (0 < ν_Mo_ < 1) and the stripes are field independent. The stripes show field dispersion in region (1 < ν_Mo_ < 2) but are still substantially different from the layer-decoupled case, indicating weaker but non-negligible interlayer hybridization. Furthermore, the mixed valence topological insulator (MVTI) exhibiting the quantum spin Hall effect at ν = 2 and the Chern insulator at ν = 1 (in the presence of strong Coulomb interactions^31–35,52,53^ ) require interlayer hybridization and are observed only in AA-stacked samples (Figure 4d).

## Revised Interpretation of Earlier Results

With a better understanding of the relationship between the stacking order and electronic phase diagram, we clarify the interpretation of several earlier studies. The first concerns the nature of the Chern insulator at ν = 1. An earlier study assigned the samples as AB-stacked bilayers based on the uncalibrated SHG.^29^ By studying the layer-resolved MCD response, ref.^54^ further concluded that the Chern state is valley coherent. Based on the corrected stacking order assignment and the reported layer-resolved MCD results,^54^ we conclude that the Chern insulator is a valley-polarized state, which is consistent with most theoretical expectations.^31–35^

The second is the metal–insulator transition induced by electric field at fixed filling ν = 1 (ref 1). The transition was thought to occur in AA-stacked samples based on the uncalibrated SHG. The metal–insulator transition was interpreted as a bandwidth-tuned Mott transition because electric field in AA-stacked bilayers can tune the moiré bandwidth like in small-angle twisted homobilayers.^52,53,55–66^ With the revised stacking order assignment, we note that moiré bandwidth tuning by electric field is unlikely due to the negligible interlayer hybridization in AB-stacked bilayers. But electric field can tune the charge transfer gap between the WSe_2_ moiré band and the MoTe_2_ Hubbard band through the linear interlayer Stark effect. The charge transfer gap, acting as a Mott gap, is continuously tuned to zero at the metal–insulator transition; such a transition belongs to the same universality class as the bandwidth-tuned Mott transition.^67^

The last concerns the Kondo lattice phenomena observed in region (1 + ν_W_) of the phase diagram. The samples were assigned as AB-stacked bilayers based on the uncalibrated SHG previously,^20^ but their negligible interlayer hybridization would imply a negligible Kondo coupling. The difficulty is resolved by the revised stacking order assignment: the finite interlayer hybridization in AA-stacked samples produces a measurable Kondo coupling between the local moments in the MoTe_2_ layer and the itinerant holes in the WSe_2_ layer. The arc-shaped boundary in the MCD response under a fixed *B*-field (Figure 4c) separates a light Fermi liquid and a heavy Fermi liquid as reported in ref.^51^ To the left of the boundary, the Zeeman field overcomes the Kondo coupling, the local moments and the itinerant holes are decoupled, and the polarized local moments give a strong MCD response. To the right of the boundary, the local moments and the itinerant holes are coupled to form Kondo singlets, resulting in a weak MCD response. The shape of the boundary is given by the dependence of Kondo coupling on the filling factor and band alignment (or electric field). In contrast, the strong MCD or local moment response is observed throughout region (1 + ν_W_) for AB-stacked bilayers (Figure 3c).

In summary, stacking order plays a crucial role in the electronic properties of TMD moiré bilayers because interlayer hybridization is stacking-order dependent. In this study, we calibrated the optical SHG—a commonly used technique for the stacking order characterization—by atomic resolution imaging using the STEM. Once the calibration is established, only SHG characterization is required to determine the stacking order of MoTe_2_/WSe_2_ moiré bilayers in future studies. We also directly correlate the stacking order with the transport and magnetic properties and the electronic phase diagram of the material. With the revised stacking order assignment, we resolved difficulties in the interpretation of earlier results, including the nature of the Chern insulator, mechanism of an electric-field-tuned metal–insulator transition at half band filling, and the Kondo lattice physics. Our work highlights the importance of calibrating SHG by STEM in determining the stacking orders of angle-aligned TMD heterobilayers in general; it also establishes an experimental approach for correlating SHG and STEM characterizations for generic air-sensitive layered heterostructures.

## Supplementary Material



## Notes

Methods for STEM and Hall bar device fabrication; STEM, SHG, electrical transport, and magneto-optical measurement procedures; stacking-dependent exciton hybridization; supplementary figures on atomic-resolution imaging, stacking-order determination, SHG wavelength dependence, exciton hybridization, and zero-field transport and MCD (PDF)
