# Supplementary material for: Stacking-Order-Dependent Electronic Properties of MoTe2/WSe2 Moiré Bilayers
Source: Nano Lett. 2026 Oct 26;26(43):14829–36. doi: 10.1021/acs.nanolett.6c02425 (PMC13741919; doi:10.1021/acs.nanolett.6c02425)
Supplement: Supplementary file 1 [file nl6c02425_si_001.pdf]

Supporting Information for "Stacking-Order-Dependent Electronic Properties of $MoTe_2/WSe_2$ Moiré Bilayers"

Zhongdong Han[1,2,3], Wenjin Zhao[4], Eegene Clara Chung[2,5], Chia-Hao Lee[5], Zui Tao[5], Zhengchao Xia[5], Yichi Zhang[2], Yiyu Xia[5], Jekwan Lee[1,2,3], Bowen Shen[5], Ariana Ray[2], Yu-Tsun Shao[5], Tingxin Li[5], Shengwei Jiang[1,2], Yihang Zeng[1,2], Kenji Watanabe[6], Takashi Taniguchi[6], David A. Muller[4,5], Kin Fai Mak[1,2,3,4,5†], Jie Shan[1,2,3,4,5†]

[1]Laboratory of Atomic and Solid State Physics, Cornell University, Ithaca, NY, USA
[2]Department of Physics, Cornell University, Ithaca, NY, USA
[3]Max Planck Institute for the Structure and Dynamics of Matter, Hamburg, Germany
[4]Kavli Institute at Cornell for Nanoscale Science, Ithaca, NY, USA
[5]School of Applied and Engineering Physics, Cornell University, Ithaca, NY, USA
[6]National Institute for Materials Science, Tsukuba, Japan

[†]Email: kin-fai.mak@mpsd.mpg.de; jie.shan@mpsd.mpg.de

Methods

STEM sample fabrication

Samples for the scanning transmission electron microscopy (STEM) measurements were fabricated using the layer-by-layer dry transfer method[1]. In short, $MoTe_2$ and WSe_2 monolayers were exfoliated from bulk crystals onto 285-nm SiO_2/Si substrates and identified by their reflection contrast. Polarization-resolved optical second harmonic generation (SHG) was performed on each monolayer; the second harmonic (SH) intensity exhibits a six-fold rotational symmetry, revealing the transition metal dichalcogenide (TMD) crystallographic orientation. Each TMD monolayer was then cut into two halves using an AFM (atomic force microscopy) tip and two $MoTe_2/WSe_2$ bilayers with distinct stacking orders were assembled using one half from each flake. In both samples, the two TMDs were angle aligned according to their SHG, resulting in either parallel (AA) or antiparallel (AB) stacking order. A 180° rotation was introduced in the two halves of $MoTe_2$.

The heterostructures were assembled using the dry transfer method. First, a thin (3-5 nm) hexagonal boron nitride (hBN) flake was picked up by a polycarbonate (PC) film on a polydimethylsiloxane (PDMS) stamp. Next, the TMD flakes were picked up sequentially with the desired angle alignment. Last, the sample was covered by a thin (~ 1 nm) graphite capping layer to prevent oxidation of $MoTe_2$. All processes involving $MoTe_2$ prior to encapsulation were carried out in a nitrogen environment to minimize degradation.

Each sample has three distinct regions: two isolated monolayer regions and a bilayer region (Fig. 2a,b). Polarization-resolved SHG was performed on each region of the sample on the stamp. It was then released onto a TEM grid (PELCO 21589-10, silicon nitride membranes with holes). Annular dark-field STEM measurements were carried out on the same three regions to determine the lattice structure of the monolayers and their stacking order in the bilayer. Such a sample design enabled a direct correlation of SHG with the stacking order.

Hall bar device fabrication

Dual-gated Hall bar devices of angle-aligned $MoTe_2/WSe_2$ bilayers for combined electrical transport and optical measurements were fabricated using the method reported in previous studies[2,3]. Thin flakes required for device assembly—including few-layer graphite and hBN as gate electrodes and dielectrics, as well as monolayer TMDs—were exfoliated from bulk crystals onto 285-nm SiO_2/Si substrates and identified by their reflection contrast. Polarization-resolved SHG (calibrated by STEM above) was used to determine the crystallographic orientation of the TMD monolayers, angle align them and determine the stacking order of the bilayers.

A two-step stacking process was used to fabricate high-quality devices. First, hBN and graphite flakes were picked up and released onto a Si substrate with prepatterned electrodes to form the bottom gate. Thin (8 nm) platinum electrodes were deposited onto the hBN to define a Hall bar geometry using the standard electron-beam lithography and evaporation techniques. Next, the polymer residues on the device were removed by the AFM in the contact mode with a typical force of 500 nN. Last, the rest of the device, consisting of a graphite/hBN top gate and an angle-aligned $MoTe_2/WSe_2$ bilayer, was released onto the bottom-gate substrate. All processes involving $MoTe_2$ prior to encapsulation were carried out in a nitrogen environment to minimize oxidation. In these devices, the bottom gate fully covers the top gate, thereby eliminating parallel conduction channels, and the top-gate hBN dielectric[4] is substantially thinner (< 4 nm) than that in typical devices. Such a design enabled the application of high (up to 1.4 V/nm) electric fields perpendicular to the sample plane.

STEM measurements

STEM images were collected on two Thermo Fisher Spectra 300 aberration-corrected microscopes with an ultrahigh brightness cold field emission gun (X-CFEG). The acceleration voltage was 120 kV to minimize beam-induced damage. The probe-semi-convergence angle was 30 mrad with the probe current between 35-55 pA. The dwell times were 0.5 μs for 10-frame imaging and 5-10 μs for single frame imaging. The multi-frame images were rigidly registered to mitigate scan distortions. Annular dark-field (ADF) images were acquired with inner and outer collection angles of 48 to 200 mrad to balance signal intensity with atomic Z-contrast. The pixel sizes ranged from 5.7 to 21 pm. The measurements were performed with samples at room temperature.

Second harmonic measurements

The optical SH measurements were performed in the reflection geometry using normal incidence excitation at room temperature. The output of a mode-locked Ti:sapphire oscillator (Spectra Physics, Tsunami) operating at an 80 MHz repetition rate was used as the excitation source. The pulses were of 100 fs duration and centered at 800 nm. The excitation beam passed through a linear polarizer and an achromatic half-wave plate and was focused onto a spot size of about 1 μm on the sample by an objective with numerical aperture 0.6. The retroreflected SH signal (400 nm) was collected by the same objective, passed through the same half-wave plate, and was separated by a beam splitter and filtered by a short-pass filter. An analyzer was used to select the polarization component that is perpendicular to the polarization of the excitation beam. The SH radiation was detected by an EMCCD (electron-multiplying charge-coupled device) camera (ANDOR iXon^{EM}+). The SH character was verified by its quadratic intensity dependence on the

excitation intensity. The orientational dependence of the SH response was measured by rotating the achromatic half-wave plate, which is equivalent to rotating the sample.
To minimize effects of sample degradation and heating, we kept the optical excitation power substantially below 1 mW; we also kept the sample in an inert gas (nitrogen) environment during the measurement. The SHG intensities from both TMD layers showed no observable change with time, supporting the absence of sample degradation. The observed degree of constructive (destructive) interference in SHG in the AB- (AA-) stacked samples is also independent of the excitation power in the low excitation power limit. In contrast, we observed irreversible sample damage, accompanied by an abrupt loss of the SHG signal, under substantially higher excitation powers. We kept our measurements far from this limit.

Polarization-resolved SHG has been employed to determine the crystal symmetry and crystallographic orientation of TMDs[5-7]. Monolayer TMDs (D_{3h} symmetry) lacking inversion symmetry allow for a second-order response. The second-order susceptibility tensor $\chi^{(2)}$ contains one independent nonvanishing element, $\chi^{(2)}_{\mathrm{xxx}} = -\chi^{(2)}_{\mathrm{xyy}} = -\chi^{(2)}_{\mathrm{yxy}} = -\chi^{(2)}_{\mathrm{yyx}}$, where x and y axes correspond to the armchair and zigzag directions of the crystal, respectively. Excitation radiation with electric-field amplitude E at the fundamental frequency induces a second-order nonlinear polarization $P^{(2)} \sim \chi^{(2)}: EE$, which contains components at the SH frequency. The SH intensity in cross polarization is expected to exhibit a six-fold symmetric response as a function of the crystal's azimuthal angle (or the linear polarization angle of the excitation beam), where the maxima (minima) correspond to the zigzag (armchair) directions of the crystal. In angle-aligned bilayers, each layer generates an SH response which adds constructively or destructively, depending on the stacking order. This effect is particularly pronounced in natural homobilayers (with AB stacking order), where inversion symmetry is restored and the SH response vanishes. The method has not been verified for generic TMD heterobilayers.

Electrical measurements
Electrical transport measurements were performed in a closed cycle ^{4}He cryostat (Oxford TeslatronPT) with magnetic fields up to 14 T and temperatures down to 1.5 K. Four-terminal resistance (R_{xx}) and Hall resistance (R_{xy}) were measured using the standard low-frequency lock-in techniques at 13.77 Hz with 1 mV AC excitation applied to the source electrode. Voltage drop between the probe electrodes and source-drain current were simultaneously recorded. All data were taken at 1.6 K unless otherwise specified.

Magneto-optical measurements and analyses
Reflective magnetic circular dichroism (MCD) using normal incidence excitation was used to study the magnetic properties of the samples. Measurements were performed in a closed cycle ^{4}He cryostat (attocube attoDry 2100) with magnetic fields up to 9 T and temperatures down to 1.6 K. A superluminescent light emitting diode (SLED) with broadband emission centered at 1050 nm was used as the excitation source. The SLED output was coupled into a single-mode fiber, collimated using a 10× objective, and focused onto a spot size of 1 μm on the sample using a low-temperature objective with numerical aperture 0.8. The incident power on the sample was kept below 30 nW to minimize the heating effects. The reflected light was collected by the same objective and detected by a spectrometer coupled with a liquid-nitrogen-cooled CCD (charge-coupled device). A combination of a linear polarizer and an achromatic quarter-wave plate was used to generate circularly polarized light. The MCD spectrum, $(R^+ - R^-)/(R^+ + R^-)$, was

obtained by comparing the reflection spectra R^+ and R^- for left and right circularly polarized light, respectively. The spectrally averaged MCD over the attractive polaron resonance of $MoTe_2$ is illustrated in Figs. 3c, 4c and S7. It was obtained by averaging the absolute value of MCD over a wavelength range of 1068-1128 nm for AA-stacked samples and 1050-1120 nm for AB-stacked samples. All data were taken at 1.6 K unless otherwise specified.

Stacking-dependent exciton hybridization

Intralayer and interlayer optical transitions in $MoTe_2/WSe_2$ bilayers that share the same final (or initial) states can hybridize when the interlayer resonance is tuned by electric field to cross the intralayer resonance. Supplementary Fig. S5 illustrates the reflection contrast (RC) spectrum of $MoTe_2/WSe_2$ bilayers near the WSe_2 fundamental exciton resonance as a function of electric field at fixed filling factor $\nu = 1$. The RC spectrum, $(R - R_0)/R_0$, was obtained by comparing the raw reflection spectrum R to a reference spectrum R_0 (which was measured at a high doping density with quenched exciton resonances). Hybridization between the intralayer and interlayer excitons is shown as level repulsion. The slope of the exciton energy as a function of the electric field (which is approximately the shift per unit electric field of the interlayer potential difference) corresponds to a dipole moment of approximately $0.28\ \mathrm{e} \cdot \mathrm{nm}$. The value is consistent with $\frac{\varepsilon_{\mathrm{hBN}}}{\varepsilon_{\mathrm{TMD}}} et$, where $\varepsilon_{\mathrm{hBN}}$ ~ 3 and $\varepsilon_{\mathrm{TMD}}$ ~ 7 are the out-of-plane dielectric constants of hBN and TMD, respectively, and t ~ 0.7 nm is the interlayer distance. Exciton hybridization is stacking-order dependent, occurring at positive fields (~ 0.3 V/nm) for AA-stacked samples and negative fields (~ -0.3 V/nm) for AB-stacked samples (a positive electric field shifts the energy bands of WSe_2 up relative to those of $MoTe_2$). They correspond to an interlayer potential difference of about 100 meV and -100 meV for the AA- and AB-stacked samples, consistent with a spin splitting of ~ 200 meV for the valence bands in $MoTe_2$.

The stacking-dependent exciton hybridization in $MoTe_2/WSe_2$ can be understood by considering the schematic energy band diagram (Supplementary Fig. S5a,b). The bilayers are a type I heterojunction with the $MoTe_2$ band gap lying entirely inside the WSe_2 band gap. The electronic states near the band edges are the K-valley states, which are split by the Ising spin-orbit coupling. In the AA-stacked samples, the spin-up valence bands are above the spin-down bands in both TMDs, whereas in the AB-stacked samples, the order of the two spin-split bands is reversed in $MoTe_2$. Since only the spin-conserving optical transitions (marked by arrowed lines) are dipole allowed, level repulsion is expected at a higher E-field in AA-stacked samples. This is fully consistent with experiment. The values of the E-fields for hybridization are dependent on the band offset and the difference in the intralayer and interlayer exciton binding energies. The topmost valence bands of $MoTe_2$ and WSe_2 align at ~ 0.7 V/nm, whereas exciton hybridization occurs near 0.3 V/nm in AA-stacked samples. With an effective dipole moment of $0.28\ \mathrm{e} \cdot \mathrm{nm}$, this field difference implies that the intralayer exciton binding energy is about 100 meV larger than the interlayer exciton binding energy.

References

1 Wang, L. *et al.* One-dimensional electrical contact to a two-dimensional material. *Science* **342**, 614-617 (2013).

2 Li, T. *et al.* Continuous Mott transition in semiconductor moiré superlattices. *Nature* **597**, 350-354 (2021).
3 Li, T. *et al.* Quantum anomalous Hall effect from intertwined moiré bands. *Nature* **600**, 641-646 (2021).
4 Han, Z. *et al.* Topological Kondo insulator in $MoTe_2$/WSe_2 moiré bilayers. *Nature Physics*, **22**, 396-401 (2026).
5 Li, Y. *et al.* Probing symmetry properties of few-layer MoS_2 and h-BN by optical second-harmonic generation. *Nano Letters* **13**, 3329-3333 (2013).
6 Malard, L. M., Alencar, T. V., Barboza, A. P. M., Mak, K. F. & De Paula, A. M. Observation of intense second harmonic generation from MoS_2 atomic crystals. *Physical Review B* **87**, 201401 (2013).
7 Kumar, N. *et al.* Second harmonic microscopy of monolayer MoS_2. *Physical Review B* **87**, 161403 (2013).

Supplementary figures

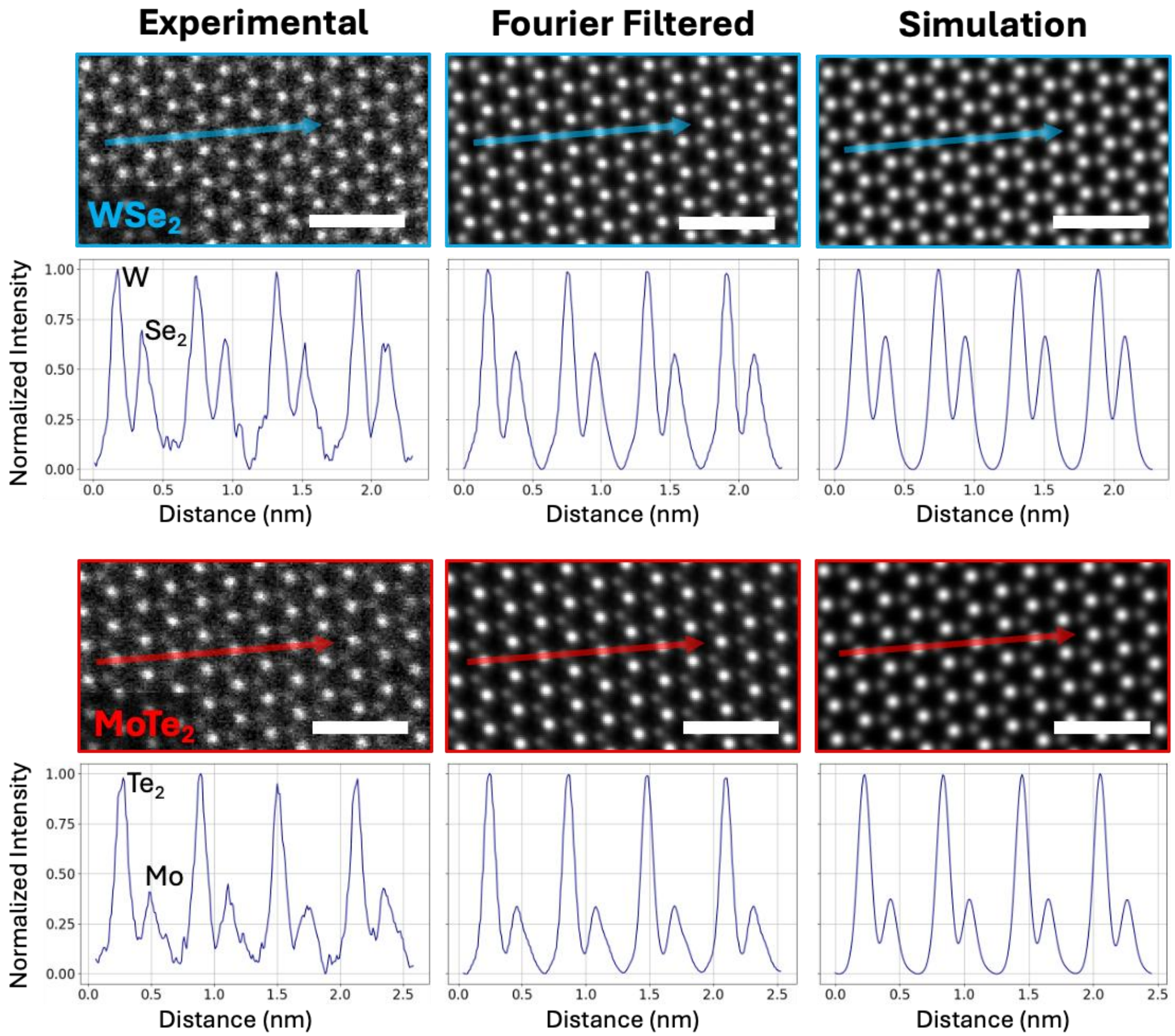

Supplementary Figure 1. Atomic-resolution imaging and intensity profile analysis. Top panels display experimental (raw), Fourier-filtered, and simulated ADF-STEM images for WSe_2 (blue) and $MoTe_2$ (red) monolayers. Corresponding normalized intensity line profiles (bottom) are extracted along the indicated colored arrows. Accurate atomic species identification is confirmed by the excellent agreement between experimental, Fourier filtered, and the simulated images. All scale bars are 1 nm.

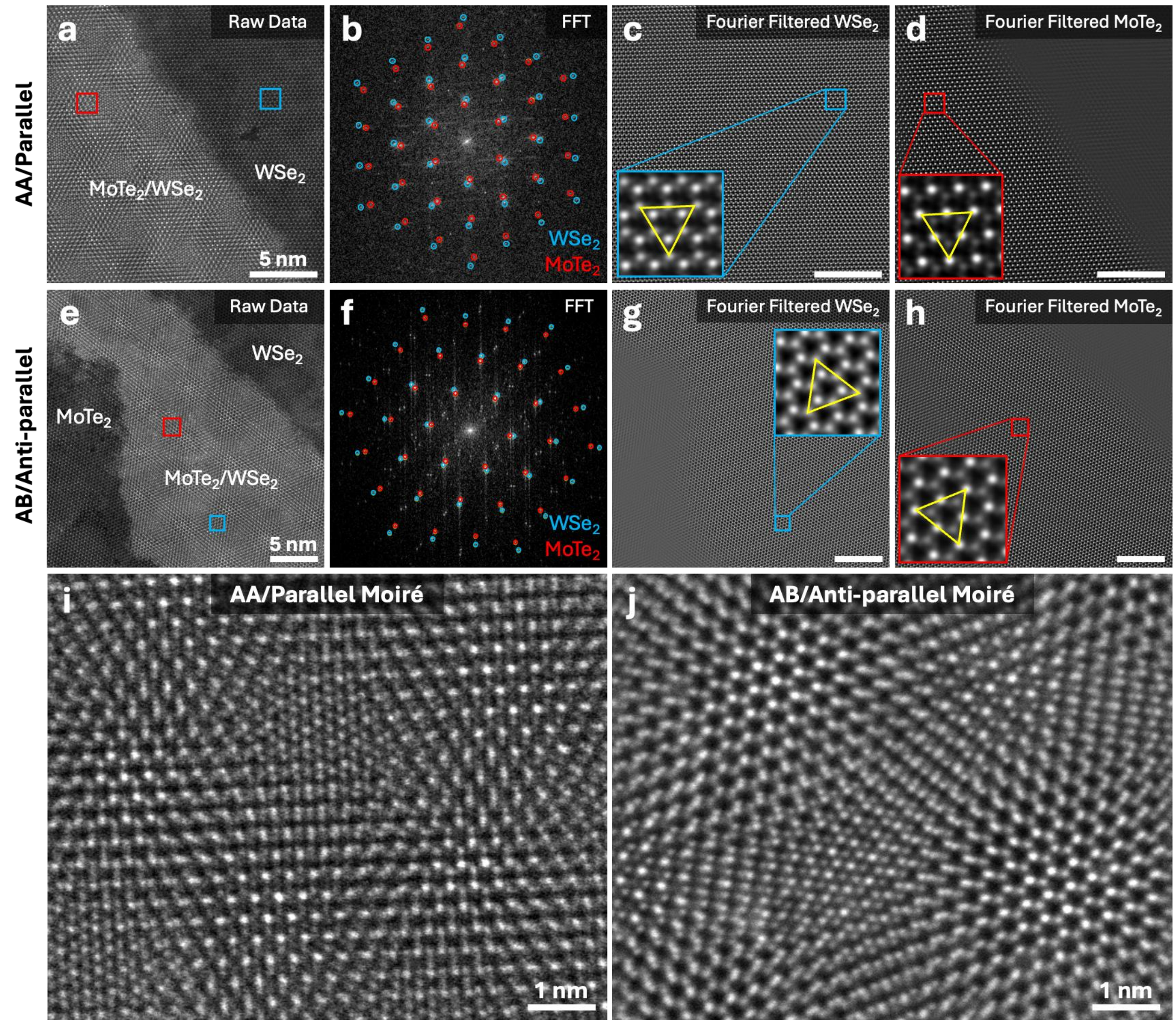

Supplementary Figure 2. Determination of stacking order of $MoTe_2/WSe_2$ bilayers via ADF-STEM and Fourier filtering. a–d, AA/parallel stacking. Raw ADF-STEM image (**a**) showing the bilayer region and isolated WSe_2 region. Fast Fourier transform (FFT) of the bilayer region (**b**), where blue and red circles denote the reciprocal lattice points for WSe_2 and $MoTe_2$, respectively. Fourier-filtered images of the individual WSe_2 (**c**) and $MoTe_2$ (**d**) lattices. Insets show magnified views with yellow triangles indicating the orientation of the metal-chalcogen sublattices. **e–h**, AB/antiparallel stacking. Scale bars in **a-h** are 5 nm. **i,j,** Moiré pattern of AA-stacked (**i**) and AB-stacked (**j**) bilayers. Scale bars in **i,j** are 1 nm. Determining stacking order via moiré analysis is difficult hence isolated monolayer regions are investigated.

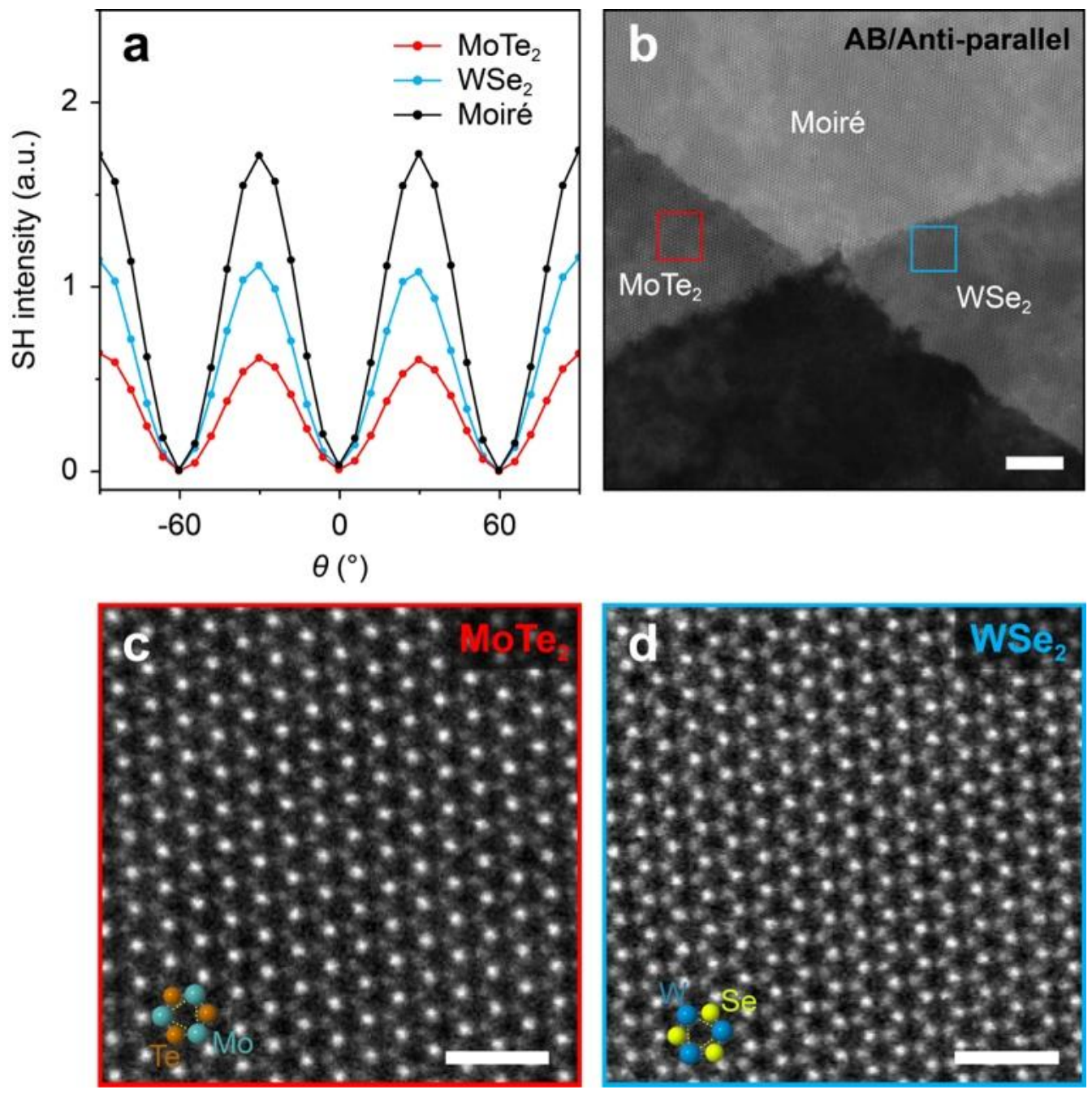

Supplementary Figure 3. SHG and ADF-STEM characterization on another AB-stacked (antiparallel) sample. a, SH intensity as a function of the polarization angle for excitation at 800 nm from the bilayer (moiré) region and two isolated monolayer regions. The bilayer region exhibits a significantly enhanced SH response. **b,** Large field-of-view ADF-STEM image covering both the monolayer and the bilayer regions. The scale bar is 5 nm. **c,d,** Zoom-in views of monolayer $MoTe_2$ (**c**) and WSe_2 (**d**). Scale bars in **c,d** are 1 nm.

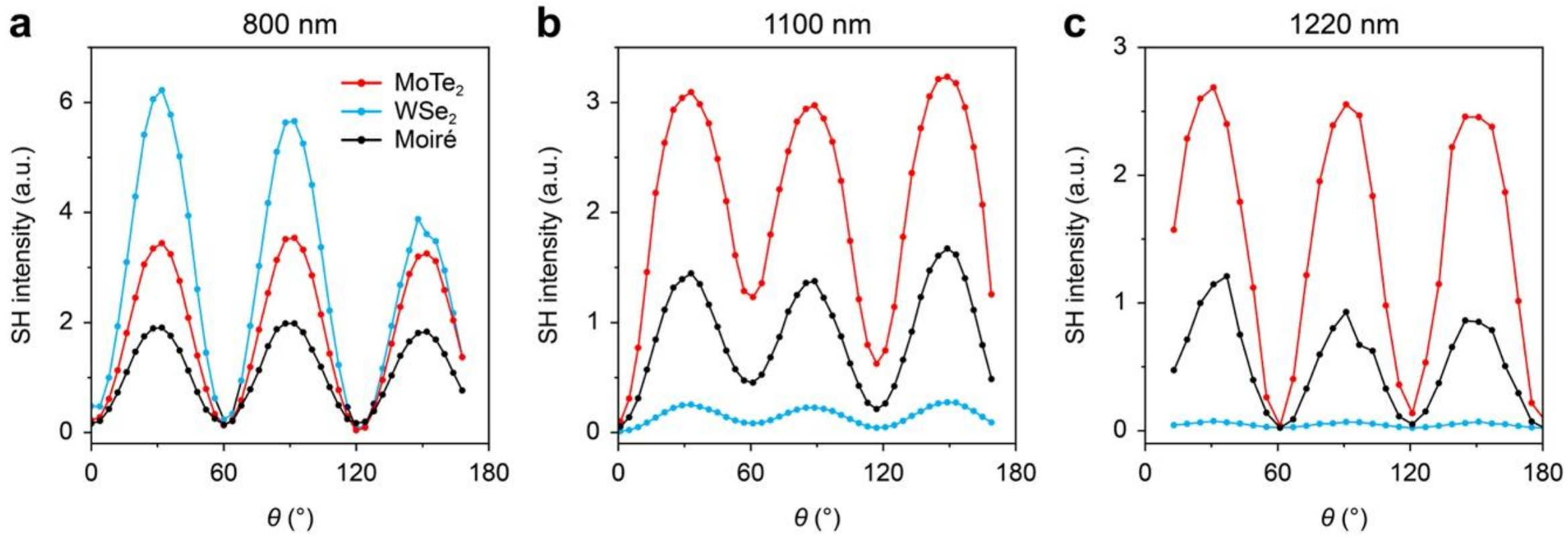

Supplementary Figure 4. Wavelength dependence of the optical SHG. a-c, SH intensity as a function of the excitation polarization angle (Methods) obtained from an AA-stacked $MoTe_2/WSe_2$ bilayer on a Si substrate. The SHG from the bilayer (moiré) region and the isolated monolayer regions depends on the excitation wavelength. It is also sensitive to sample quality and doping density. Destructive interference of the SH radiation in the bilayer is observed for all three excitation wavelengths (800 nm (**a**), 1100 nm (**b**), and 1220 nm (**c**)), with most complete cancelation at 800 nm. Compared to Fig. 2f, the destructive interference is less complete under 800 nm excitation (**a**); it is correlated with a bigger difference in the SH intensities from the two constituent TMD layers; the difference is likely caused by the different sample quality and unintentional doping densities. The bilayer intensity is also less than that of monolayer $MoTe_2$ under 1220 nm excitation (**c**); it is likely caused by the additional optical absorption at the SH wavelength (610 nm) from the WSe_2 layer and by the stacking-induced modifications on the $MoTe_2$ optical response. The output of an OPO (optical parametric oscillator, Coherent, Chameleon) operating at an 80 MHz repetition rate was used as the excitation source.

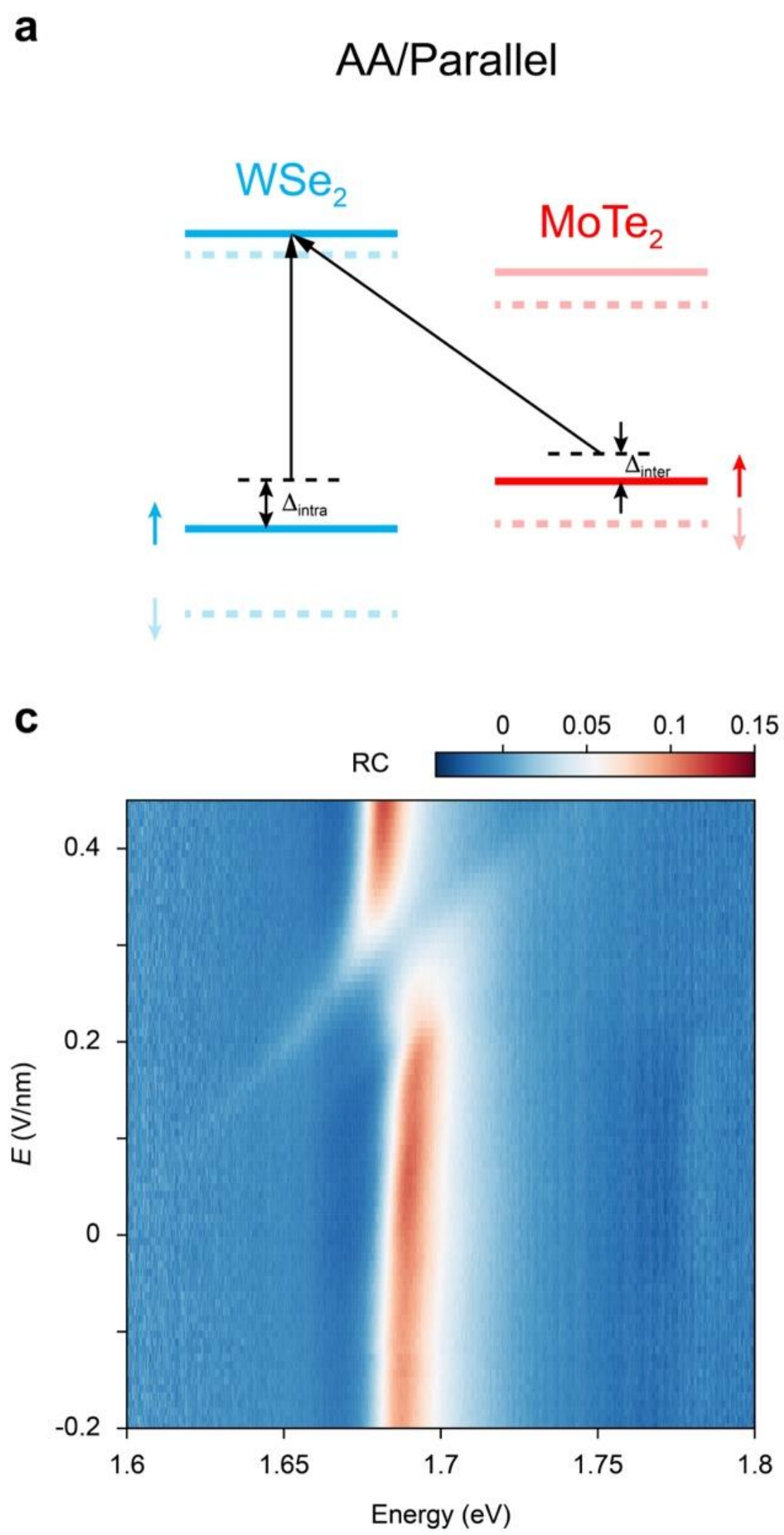

Supplementary Figure 5. Stacking-order-dependent exciton hybridization. a,b, Spin-resolved band alignment for AA-stacked (**a**) and AB-stacked (**b**) $MoTe_2/WSe_2$ under zero external electric field. The conduction and valence bands of WSe_2 (blue) and $MoTe_2$ (red) from the K valley are illustrated. The spin-up (solid lines) and spin-down (dashed lines) bands are split by the Ising spin-orbit coupling. Black arrows denote intra- and inter-layer exciton excitations in the WSe_2 layer. Only spin-conserving optical transitions are allowed; the optically inactive bands are shown in faded colors. Δ_{intra} and Δ_{inter} denote the intralayer and interlayer exciton binding energies, respectively. See Methods for details. **c,d,** Reflection contrast spectra at $\nu = 1$ as a function of E for AA-stacked (**c**) and AB-stacked (**d**) $MoTe_2/WSe_2$ bilayers.

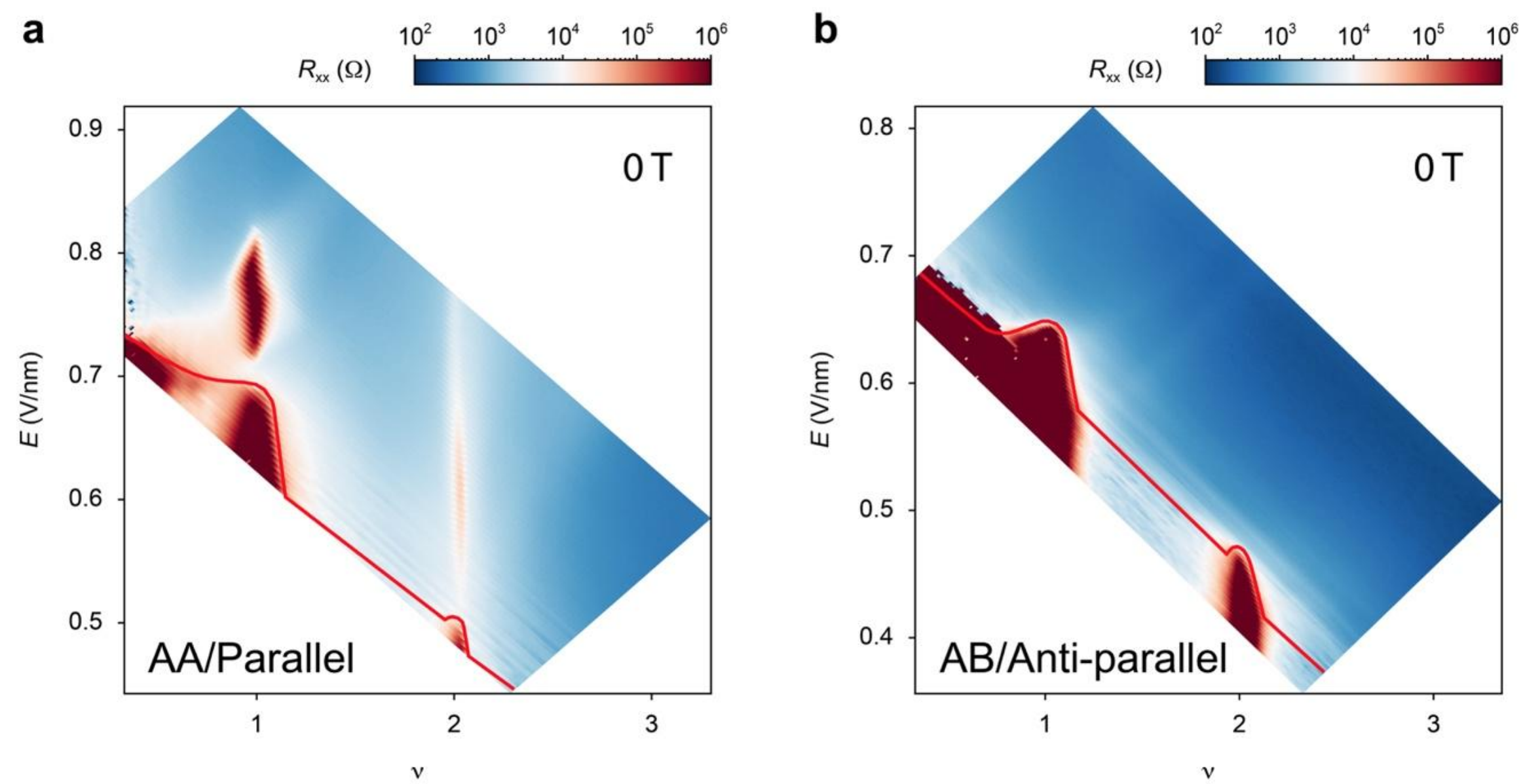

Supplementary Figure 6. Comparison of zero-field electrical transport results. a,b, Longitudinal resistance R_{xx} as a function of ν and E at $T = 1.5$ K and $B = 0$ T for AA-stacked (**a**) and AB-stacked (**b**) $MoTe_2/WSe_2$. The red curve separates the $\nu_{\mathrm{W}} = 0$ and $\nu_{\mathrm{W}} > 0$ regions. Additional insulating states at $\nu = 1$ and $\nu = 2$ in regions above the red curve are observed only in the AA-stacked sample, consistent with its substantially stronger interlayer hybridization.

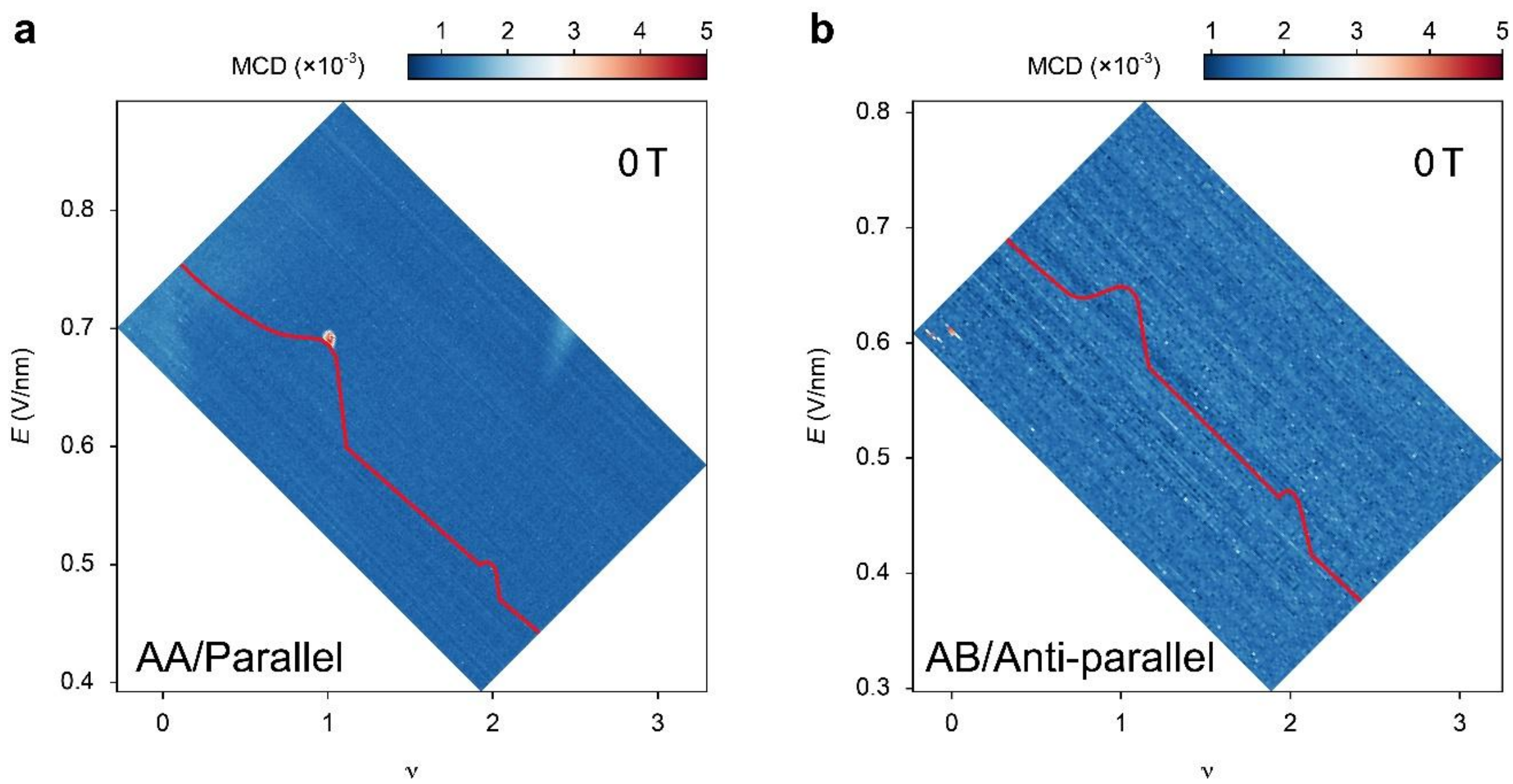

Supplementary Figure 7. Comparison of zero-field MCD results. a,b, MCD as a function of ν and E at $T = 1.6$ K and $B = 0$ T for AA-stacked (**a**) and AB-stacked (**b**) $MoTe_2/WSe_2$. The red curve separates the $\nu_W = 0$ and $\nu_W > 0$ regions. Spontaneous MCD is observed only for the Chern insulator at $\nu = 1$ in the AA-stacked sample.
